# Supplementary material for: Molecular Regulation of Yak Preadipocyte Differentiation and Proliferation by LncFAM200B and ceRNA Regulatory Network Analysis
Source: Cells. 2022 Aug 1;11(15):2366. doi: 10.3390/cells11152366 (PMC9368248; doi:10.3390/cells11152366)
Supplement: Supplementary file 1 [file cells-11-02366-s001.zip › supplement fig legend.pdf]

Figure S1. Reads length of small RNA sequencing.

Figure S2. Function annotation of DEMs target gene. The KEGG results of up-regulated DMEs targets and down-regulated DMEs targets were displayed at (**A**) and (**B**), respectively, and the GO enrichment results shown in (**C**) and (**D**). Only top 20 KEGG terms have been visualized in each result and the GO results plotted the top 10 of each category.

Figure S3. The Venn diagrams of DEGs and DEMs target genes.

Figure S4. Binding site prediction. (**A**) *LncFAM200B* and miR-6529a combination information, analyzed by RNAhybird online database (https://bibiserv.cebitec.uni-bielefeld.de/rnahybrid/). (**B**) Binding prediction of bta-miR-6529a and *TRIM21.*
